# Comparative study for analysis of carbohydrates in biological samples

**DOI:** 10.1007/s00216-021-03845-z

**Published:** 2021-12-20

**Authors:** Martin Meyer, Lidia Montero, Sven W. Meckelmann, Oliver J. Schmitz

**Affiliations:** 1 Applied Analytical Chemistry, University of Duisburg-Essen, Universitaetsstrasse 5, 45141 Essen, Germany; 2 Teaching and Research Center for Separation, University of Duisburg-Essen, Universitaetsstrasse 5, 45141 Essen, Germany

**Keywords:** Carbohydrates, Saccharides, GC–MS, LC–MS, SFC-MS, Derivatisation

## Abstract

This work presents a comparative study for the analysis of carbohydrates for four common chromatographic methods, each coupled to mass spectrometry. Supercritical fluid chromatography (SFC), hydrophilic interaction liquid chromatography (HILIC), reversed-phase liquid chromatography (RP-LC) and gas chromatography (GC) with detection by triple quadrupole mass spectrometer (QqQ-MS) are compared. It is shown that gas chromatography and reversed-phase liquid chromatography, each after derivatisation, are superior to the other two methods in terms of separation performance. Furthermore, comparing the different working modes of the mass spectrometer, it can be determined that a targeted analysis, i.e. moving from full scan to single ion monitoring (SIM) and multiple reaction monitoring (MRM), results in an improvement in the sensitivity as well as the repeatability of the method, which has deficiencies especially in the analysis using HILIC. Overall, RP-LC–MS in MRM after derivatisation with 1-phenyl-3-methyl-5-pyrazolone (PMP) proved to be the most suitable method in terms of separation performance, sensitivity and repeatability for the analysis of monosaccharides. Detection limits in the nanomolar range were achieved, which corresponds to a mass concentration in the low µg/L range. The applicability of this method to different biological samples was investigated with various herbal liquors, pectins and a human glycoprotein.

**Graphical abstract:**

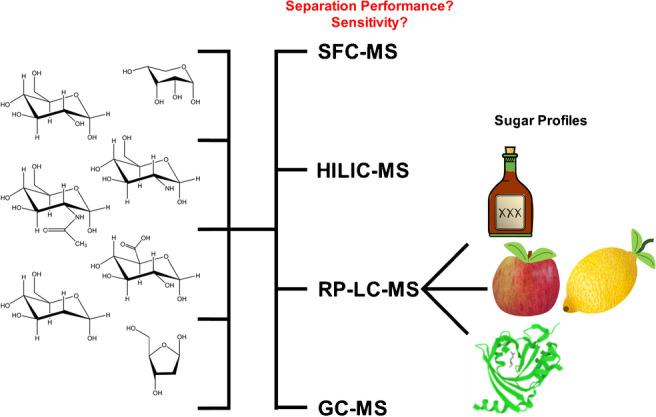

**Supplementary Information:**

The online version contains supplementary material available at 10.1007/s00216-021-03845-z.

## Introduction

Carbohydrates, also called saccharides or sugars, represent one of the most important biochemical substance classes and have a central role in the metabolism of every living organism. Thus, carbohydrates, in the form of naturally occurring or added sugars, constitute the most important dietary energy source for humans, and total sugars are consumed in varying quantities depending on the age group, between 13% for adults and 38% for infants in terms of total energy intake [1]. Carbohydrate intake further contributes to diseases and excessive or regular consumption has been associated with diabetes, dental issues and also attention deficit hyperactivity disorder (ADHD) among others [2–4]. However, carbohydrates also have major importance aside from nutrition and food science as constitutional components of di-, oligo- and polysaccharides. These are, for example, structural and stabilising elements of extracellular polymeric substances in microbial biofilms [5], parts of glycoproteins, which are essential in the immune response of the human body [6] or represent the glycosidic residue in the substance class of flavonoids, having antioxidant properties [7]. Thus, carbohydrates represent an important class of substances to be investigated in many research disciplines, such as food chemistry, clinical research, bioanalytics and many more. A large number of analytical methods have been published over the years, which vary depending on the field of application and the explicit objective. First of all, it has to be determined in which manner the carbohydrates are to be analysed, i.e. whether the monomeric composition is to be investigated or more complex chained or branched polymers, or even glycosidically bound to other molecules.

The analysis of sugar monomers in samples can be carried out using various chromatographic methods. Liquid chromatographic (LC) methods are used to take advantage of the various available stationary phases. Separation of sugars has been carried out using amino and amide as well as HILIC phases, which separate the carbohydrates in their native form without derivatisation [8–10]. Other LC methods used for the separation of sugars are based on ionic interactions, such as high-performance anion exchange chromatography often coupled with pulsed amperometric detection (HPAEC-PAD), especially used in the field of bioanalytics [11], or require prior derivatisation to make the generally highly polar monosaccharides accessible for reversed-phase liquid chromatography (RP-LC). In RP-LC applications, monosaccharides are first derivatised for example with 1-naphthylamine [12] or 1-phenyl-3-methyl-5-pyrazolone [13]. The subsequent analysis is carried out using C18 stationary phases with which no separation of the saccharides would be expected otherwise. Alternatives to LC analysis, which are also commonly applied for the analysis of monomeric compositions, are capillary electrophoresis (CE) [14], as well as gas chromatography (GC). Using the latter technique, derivatisation is also necessary due to the thermal instability and low volatility of saccharides. Different derivatisation approaches, including silylation or methylation, among others, have been established and are applied in the GC analysis of carbohydrates [15]. As supercritical fluid chromatography (SFC) is currently undergoing a revival, this hybrid technique, which can be categorised somewhere in between LC and GC, is also being used in the field of metabolomics and thus also for the analysis of polar metabolites such as carbohydrates. The advantages of SFC are that it is similar to normal phase techniques, and therefore no derivatisation is necessary. Furthermore, the use of CO_2_ as a mobile phase reduces the environmental impact [16–18].

Detection of the separated carbohydrates is performed with common detectors for the respective chromatographic method. Mass spectrometers (MS) are frequently used as universal detectors for LC and GC, and flame ionisation detectors (FID) for GC and SFC. For CE and LC, UV/Vis detectors are limited because carbohydrates lack a chromophore and thus require the prior addition of a chromophore by pre- or postcolumn derivatisation [19, 20] or oxidation [14] in order to be detected using those detectors. Moreover, indirect detection can be applied [21]. Fluorescence detectors are also applicable but labelling with fluorescence markers is necessary to improve the sensitivity of detection as well [12]. In addition, detection by refractive index (RI) is also a frequently used method but it is limited to isocratic separations [22].

Analysis of more complex carbohydrates without full cleavage into monomeric units by, e.g. acid hydrolysis, such as glycosidically bound saccharides, polysaccharides or glycoproteins, requires non-target approaches and complex workflows to determine size, sequence and linkages. Size exclusion chromatography/gel permeation chromatography (SEC/GPC) and matrix-assisted laser desorption/ionisation coupled to mass spectrometry (MALDI-MS) are commonly used for size determination [23, 24]. For the sequencing of more complex saccharides, available methods comprise fragmentation studies using tandem mass spectrometry (MS/MS) [25] or ion mobility mass spectrometry (IM-MS) [26, 27]. For non-target analysis of glycosidically bound saccharides, LC-IM-MS coupling has been applied as well; in this case, the identification of analytes in complex samples was performed using database matching [28].

Given the vast number of published and applied methods, there is no “gold standard” in the analysis of carbohydrates, so that the choice of analytical method depends on the sample as well as the actual objective. In this comparative study, four methods based on different chromatographic techniques and separation mechanisms, each coupled to mass spectrometry, will be directly assessed regarding sensitivity, as well as applicability as a routine method in terms of standard deviation for the determination of the monomeric composition of a saccharide sample. For this approach, the different separation techniques were used to screen a set of monosaccharides commonly found in nature and food, which were selected as they are among the most common naturally occurring monosaccharides in order to cover the maximum of real applications, and the methods were evaluated regarding their separation performance and sensitivity in different modes of the QqQ mass spectrometer. After finding the most suitable method, it was applied to a number of biological samples to assess their sugar profiles. With this, we provide a better understanding of the advantages and disadvantages of different methods for the analysis of carbohydrates by directly comparing them with the same conditions by means of choice of target analytes and concentration ranges. To best of our knowledge, there is no comparable study to our approach.

## Materials and methods

### Chemicals and samples

d-Galacturonic acid (GalA) (≥ 97%) was purchased from Fluka (Buchs St. Gallen, Switzerland). 2-Deoxy-d-glucose (2dGlc), 2-deoxy-d-ribose (dRib), d-galactosamine (D-GalN), d-glucose (D-Glc), d-glucuronic acid (GlcA), d-mannose (D-Man), d-xylose (D-Xyl), l-fucose (L-Fuc), l-glucose (L-Glc), l-rhamnose (L-Rha), N-acetyl-d-galactosamine (GalNAc) and N-acetyl-d-glucosamine (GlcNAc) (each min. ≥ 95%) were purchased from Carl Roth (Karlsruhe, Germany). d-Galactose (D-Gal), d-glucosamine (D-GlcN), d-ribose (D-Rib) and l-arabinose (L-Ara) were obtained from Sigma-Aldrich (St. Louis, USA). Carbon dioxide (CO_2_) (N48, 99.998%) was purchased from Air Liquide (Düsseldorf, Germany). Ammonia solution (25%, Suprapur) and ammonium acetate in LC–MS grade were obtained from Merck (Darmstadt, Germany); 1-phenyl-3-methyl-5-pyrazolone (PMP) (99%) and ammonium formate of LC–MS grade were purchased from Sigma-Aldrich (St. Louis, USA); acetonitrile and methanol, each LC–MS grade, and glacial acetic acid in HPLC grade were obtained from VWR International (Darmstadt, Germany). Methoxamine hydrochloride (MeOX), N,O-bis(trimethylsilyl)trifluoroacetamide (BSTFA) and pyridine (each min. ≥ 98%) were purchased from Sigma-Aldrich (St. Louis, USA). Ultrapure and desalted water with a resistivity of 18.2 MΩ∙cm was prepared by a Sartorius Stedim water purification system (Göttingen, Germany).

As application of the optimised method, eight herbal liquors were used. They were purchased in local supermarkets in 2019; the samples were AA (29% vol. ethanol), BN (31% vol. ethanol), JR (35% vol. ethanol), KG (35% vol. ethanol), KH (42% vol. ethanol), RI (30% vol. ethanol), SN (30% vol. ethanol) and UG (44% vol. ethanol).

Furthermore, two pectins were analysed. Two fruit pectins, from apples and citrus peels, were purchased from Sigma-Aldrich (St. Louis, USA).

Lastly, α1-acid glycoprotein (AGP) from human plasma was analysed which was purchased from Sigma-Aldrich (St. Louis, USA).

### Sample preparation

#### Standards for method development and characterisation

For SFC and HILIC analysis, standards and samples were solved in methanol:water 50:50 (v/v) or in mobile phase B (see section “HILIC-MS analysis”), respectively. For RP-LC analysis, derivatisation was carried out using PMP, resulting in monosaccharide derivates with two PMP residues. The method was adapted and modified from the ones originally reported by Honda et al. [29] and Rühmann et al. [19], providing almost quantitative derivatisation efficiency providing only one stoichiometric product without epimerisation. Briefly, 0.1 M PMP solution in methanol and 0.4% ammonium hydroxide were mixed in volume ratio 2:1. A total of 25 µL of the sample was mixed with 75 µL of this derivatisation mixture, vortexed and centrifuged at 2000 rpm for 2 min at room temperature (MiniSpin Plus, Eppendorf, Hamburg, Germany). After that, the samples were incubated at 70 °C for 100 min. Then, 25 µL of 0.5 M acetic acid and 875 µL of ultrapure water were added. Each sample was filtrated (pore size 0.2 µM cellulose, CS—Chromatographie Service, Langerwehe, Germany) and transferred to LC vials. Samples that were not directly analysed were stored at − 20 °C until further use. For GC analysis, a prior derivatisation was also necessary. The derivatisation protocol, based on methoximation and silylation, was adapted from Fiehn [30] and optimised. Therefore, 30-µL aliquots of the samples were initially dried in a vacuum centrifuge (Concentrator plus, Eppendorf, Hamburg, Germany), then dissolved in 10 µL of a MeOX-pyridine solution (20 mg/mL) and incubated at 30 °C for 90 min. A total of 40 µL of BSTFA was added and the sample was again incubated at 37 °C for 30 min. The silylation reagent was not quenched, and this solution was immediately used for the GC analysis.

#### Herbal liquors

To obtain and differentiate sugar profiles of the eight herbal liquors, the samples were fractionated using solid-phase extraction (SPE). This approach includes the hypothesis that sugars in herbal liquors are present both unbound and glycosidically bound, for example to organic substances such as polyphenols. Since the determination of sugar profiles is desired, the cleavage of polymeric sugars as well as glycosidically bound ones through hydrolysis is necessary. Thus, after hydrolysis, it is no longer possible to distinguish between “free” and glycosidically bound saccharides. Therefore, SPE was performed on a C18 phase and two fractions were collected. Firstly, the fraction of “free” sugars [31], which includes the eluate of the sample loading and the washing step, here it is assumed, as shown by Buszewski [32], that free sugars, as very polar substances, do not show any retention on C18 phases. Secondly, the fraction of glycosidically bound sugars, presenting lower polarity and therefore higher interaction with the C18 cartridge, was collected, which corresponds to the elution step with organic solvent. The fractionation of the herbal liquors was carried out with a Chromabond C18 phase (cartridge: 6 mL, 500 mg) purchased from Macherey–Nagel (Düren, Germany). At first, 1 mL of the respective liquor was first diluted with 4 mL ultrapure water to reduce the ethanol content and thus prevent a breakthrough of the substances during sample loading. After sample loading, washing was carried out with 5 mL H_2_O/MeOH (98:2) + 0.2% FA. The eluate of the sample application and washing corresponds to fraction 1. After washing and drying of the SPE phase, elution was carried out with 5 mL ACN + 0.2% FA and the phase was dried again. This eluate corresponds to fraction 2.

#### Fruit pectins

The two pectins from apples and citrus peels were adjusted with ultrapure water to a concentration of 2 mg/mL.

#### Human glycoprotein

The α1-acid glycoprotein from human plasma was dissolved in ultrapure water to a concentration of 10 mg/L.

#### Hydrolysis

Fractionated herbal liquors, fruit pectins and the human glycoprotein samples were hydrolysed. The hydrolysis method with trifluoroacetic acid (TFA) was adapted from De Swaaf et al. [33]. First, the dry weight of all samples was determined and then 3 mg of the respective dried sample was heated with 1 mL of 2 M TFA for 3 h at 95 °C. TFA was removed; the residue was neutralised and washed with 0.5 mL of 1 M NH_4_OH solution, which was subsequently evaporated. Finally, the hydrolysed sample was dissolved in 1 mL ultrapure water and derivatised with PMP according to the protocol described above (see section “Standards for method development and characterisation”).

### Method development

The methods described below were optimised in preliminary experiments by testing different stationary phases, as well as optimising the gradient and the flow rate of the mobile phase for the highest possible separation performance, and optimising the MS parameters such as gas temperatures, collision energies and dwell times for the highest possible sensitivities.

#### SFC-MS analysis

An injection volume of 10 µL of standards and samples was analysed with an Agilent 1260 Infinity II SFC system coupled to an Agilent Ultivo Triple Quadrupole mass spectrometer (QqQ) (Agilent Technologies, Santa Clara, USA). The SFC-QqQ system comprised a 1260 SFC control module (G4301A), a 1260 SFC binary pump (G4782A), a 1260 SFC multisampler (G4767A), a 1260 MCT (G7116A), a 1260 Iso pump (G7110B) and a LC/TQ (G6465A). The separation was done using a Zorbax RX-Sil column (150 × 4.6 mm, 5 µm; Agilent, Santa Clara, USA) at constant temperature of 35 °C. The mobile phase contained CO_2_ (mobile phase A) and methanol (mobile phase B). Additionally, methanol was also used as the make-up solvent with a flow rate of 0.1 mL/min. The gradient was 10% B as initial condition, 0–10 min linear to 13.5% B, followed by 2.5 min at initial conditions for re-equilibration. The gradient time was 12.5 min at a constant flow rate of 2 mL/min.

Mass spectrometry was performed using an electrospray ionisation source (ESI) operating in positive ion mode. Nitrogen was utilized as sheath gas, drying gas and collision gas. The sheath gas and drying gas flow rates were set at 8 L/min at 100 °C and 5 L/min at 150 °C, respectively. The nebulizer was set at 10 psi and the capillary voltage and nozzle voltage were set at 5000 V and 500 V, respectively. The fragmentor and cell acceleration voltage (CAV) were set at 135 V and 9 V, respectively. The acquisition was performed as full MS, selected ion monitoring (SIM), detecting [M + Na]^+^ as most abundant ion, and on MS/MS in pseudo multiple reaction monitoring with transitions of [M + Na]^+^ to [M + Na]^+^ at 0 V collision energy using a dwell time of 200 ms. The LC system was controlled by Agilent OpenLAB CDS—Aquisition (Version 2.3). The MS system was controlled by Agilent Mass Hunter Workstation Data Acquisition (Version C.01.00).

#### HILIC-MS analysis

Standards and samples were analysed with an Agilent 1260 Infinity II LC system coupled to an Agilent Ultivo Triple Quadrupole mass spectrometer (QqQ) (Agilent Technologies, Santa Clara, USA). The LC-QqQ system comprised a 1260 Flexible pump (G7104C), a 1260 Vialsampler (G7129C), a 1260 MCT (G7116A) and a LC/TQ (G6465A). The separation was done on an AdvanceBio MS Spent Media column (100 × 2.1 mm, 2.7 µm; Agilent, Santa Clara, USA) at a constant temperature of 35 °C. Injection volume was 10 µL. The mobile phase contained 10 mM ammonium formate buffer, pH 11 (mobile phase A) and 10% 100 mM ammonium formate buffer, pH 11 in 90% acetonitrile (mobile phase B). The optimized HILIC gradient time was 18 min at a constant flow rate of 0.4 mL/min. The gradient starts at 0 min with 97% B and linearly decreased in 15 min to 89% B and in the next 0.5 min to 97% B, followed by 2.5 min at initial conditions for re-equilibration.

Mass spectrometry was performed using ESI operating in negative ion mode. Nitrogen was utilized as sheath gas, drying gas and collision gas. The sheath gas and drying gas flow rates were set at 12 L/min at 300 °C and 6 L/min at 200 °C, respectively. The nebulizer was set at 40 psi and the capillary voltage and nozzle voltage were set at 3000 V and 0 V, respectively. The fragmentor and CAV were set at 60 V and 9 V, respectively. The acquisition was performed as full MS, SIM, detecting [M-H]^−^ as most abundant ion, and on MS/MS in multiple reaction monitoring with transitions of [M-H]^−^ to [C_3_H_5_O_3_]^−^ (*m/z* 89) [34] at 0 V collision energy using optimized dwell times of 100 and 200 ms for each monosaccharide. The LC and MS systems were controlled by the software as described before.

#### RP-LC–MS analysis

PMP-derivatised samples and standards with an injection volume of 10 µL were analysed with the same Agilent 1260 Infinity II LC system coupled to an Agilent Ultivo Triple Quadrupole mass spectrometer as used for the HILIC-MS analysis. The separation was performed using a Kinetex C18 column (100 × 2.1 mm, 1.7 µm) from Phenomenex (Torrance, USA) at a constant temperature of 50 °C. The mobile phase contained 5 mM ammonium acetate buffer, pH 5.6 (Merck, Darmstadt, Germany) with 15% acetonitrile (mobile phase A) and acetonitrile (mobile phase B). The optimized gradient time was 15 min at a constant flow rate of 0.5 mL/min. The gradient started at 0% B, linear to 1% B in 2 min, increased to 5% B in 5 min and held for 2 min; linear to 18% B in 1 min, further increased to 40% B in 0.3 min and held for 2 min. Prior to the next injection, the column was equilibrated at initial conditions for 2.7 min.

Mass spectrometry was performed using ESI operating in positive ion mode. A flow of 0.5 mL/min was introduced into the ion source of the MS after 2.5 min to cut off the early eluting excess PMP. Nitrogen was utilized as sheath gas, drying gas and collision gas. The sheath gas and drying gas flow rates were set at 8 L/min at 325 °C and 6 L/min at 325 °C, respectively. The nebulizer was set at 40 psi and the capillary voltage and nozzle voltage were set at 4000 V and 500 V, respectively. The fragmentor and CAV were set at 165 V and 9 V, respectively. The acquisition was performed as full MS, SIM, detecting [M-OH + 2PMP]^+^ as the most abundant ion, and on MS/MS in multiple reaction monitoring with transitions of [M-OH + 2PMP]^+^ to [PMP + H]^+^ at optimized collision energies (20 and 30 V) for each monosaccharide using a dwell time of 100 ms. The LC and MS systems were as controlled by the software as described before.

#### GC–MS analysis

Using a split of 1:5, 1 µL of TMS-derivatised samples was analysed with an Agilent Intuvo 9000 GC system coupled to an Agilent 7010B GC/MS Triple Quadrupole mass spectrometer (Agilent Technologies, Santa Clara, USA). The GC-QqQ system comprised an Intuvo 9000 GC system (G3952A), an ALS (G4567A) and a GC/MS triple quadrupole mass spectrometer equipped with a HES EI source (G7012B). The used column was a HP-5 ms UI (30 m × 0.25 mm, 0.25 µm; Agilent, Santa Clara, USA). Hydrogen was utilized as carrier gas at a constant flow of 1.25 mL/min. The optimized gradient time was 40 min. The temperature gradient was 0–1 min isothermal at 100 °C as initial condition, 1–31 min linear to 250 °C with 5 °C/min, 31–32.9 min linear to 325 °C with 40 °C/min and 32.9–37.4 min isothermal at 325 °C, followed by cooling down to initial conditions. For the MRM analysis, the collision energy was set at 10 V with a dwell time of 100 ms.

### Data analysis and comparison

To determine the suitability of the applied chromatographic methods for analysis of carbohydrates, 17 monosaccharides commonly present in biological samples were analysed in full MS mode and detectable m/z values, as well as retention times for each component, were assigned. An important prerequisite in the selection of the monosaccharides was the possibility of chromatographic and/or mass spectrometric separation. As a criterion for the separability of the individual components, a baseline separation was chosen on the one hand, and on the other hand, in the case of co-eluting substances, a separability through mass differences. After determination of the retention times for all 17 monosaccharides, a pooled sample of the separable carbohydrates was prepared for determination of detection and quantification limits. Limits of detection (LOD) and quantification (LOQ) were determined by analysing dilutions of the pooled samples in fifteen calibration levels as triplicates (100, 80, 60, 40, 20, 10, 5, 2, 1, 0.5, 0.2, 0.1, 0.05, 0.02 and 0.01 µmol/L for each monosaccharide). LODs of the methods were calculated with minimum 3 × and LOQ with minimum 9 × signal-to-noise ratio [35, 36]. In addition, correlation coefficients *r*^2^ and the relative method standard deviation (RSD) for all individual substances in the pooled sample were determined as quality characteristics of the methods. For SFC-MS, HILIC-MS and RP-LC–MS, the measurements were carried out in full MS, SIM and MRM; for GC–MS, only retention times and m/z ratios were determined in full MS, and the analysis of the composite sample was only carried out in MRM.

For SFC, LC and GC methods, data evaluation was carried out using Agilent Mass Hunter Qualitative Analysis Navigator (Version B.08.00). EICs, SIM and MRM chromatograms were extracted from TICs using a symmetric mass tolerance of ± 0.2 m*/z*. Afterwards the peaks were integrated manually.

The methods were ultimately compared on the basis of the achieved LOD and LOQ and the separation performance by means of the separable monosaccharidic carbohydrates, as well as the applicability as a routine method in terms of given standard deviation between triplicates.

## Results and discussion

The analysis of carbohydrates presents a challenge due to their extreme polarity and variability. In samples, carbohydrates can appear as monosaccharides or more commonly as oligo- and polysaccharides, but to evaluate the native sequence of the saccharide chains, the evaluation of the monosaccharide moieties is necessary. In fact, the presence of isomers of monosaccharides is one of the most difficult analytical challenges. For these reasons, there are large numbers of methods used for the analysis of carbohydrates but there are no established methods or even analytical techniques that provide the optimal separation conditions and sensitivity parameters for the analysis of these complex analytes. For this reason, there is a need of a comparison of the most suitable analytical techniques for the determination of the quality parameters in the separation of carbohydrates to establish the best analytical technique with the highest separation performance and sensitivity. Therefore, GC, the most commonly LC separation modes, used for the separation of polar compounds, i.e. RP and HILIC, as well as the emerging SFC were compared.

### Characterisation of analytical methods

#### SFC-MS analysis

**Fig. 1:**
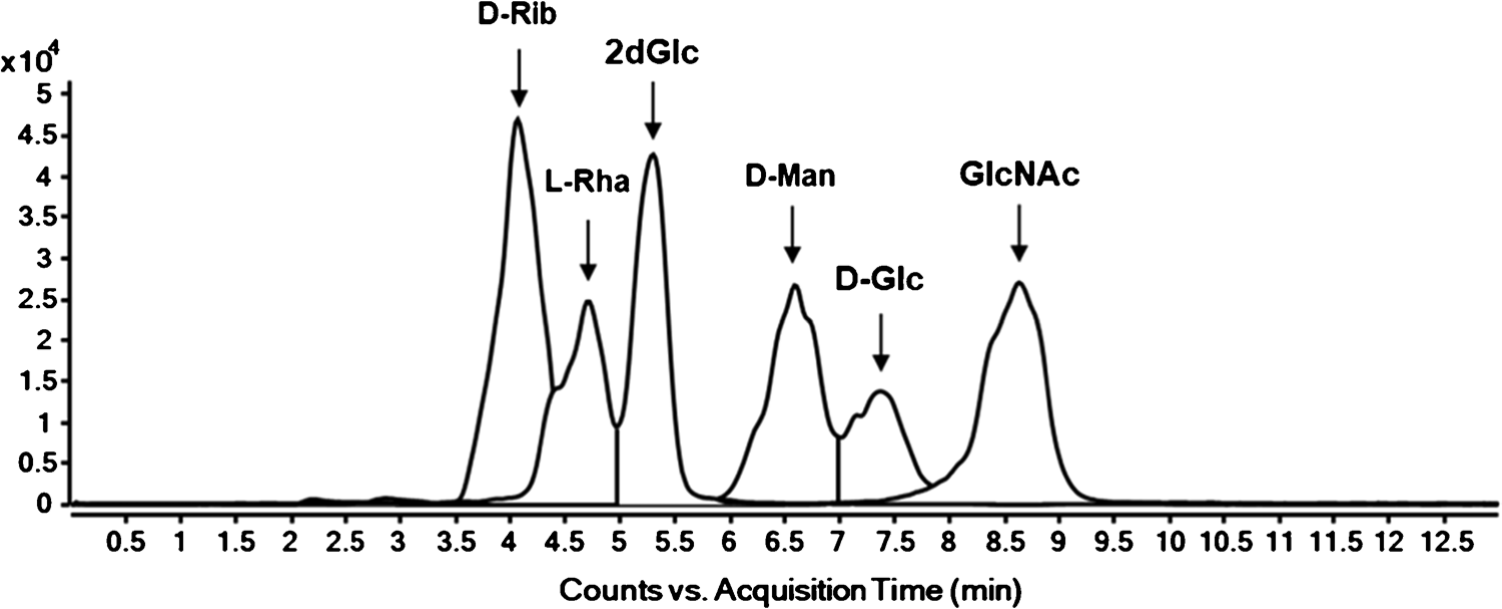
Overlaid EICs of the separable monosaccharidic species by SFC-MS (shown here: MRM)

By using SFC-MS, among the seventeen monosaccharides evaluated, the separation and identification of only six monosaccharides could be realised. Despite the high flow rate, very broad and partly asymmetrical peaks were observed. Only for D-Rib, L-Rha, 2dGlc, D-Man, D-Glc and GlcNAc distinct signals separated by chromatography or mass spectrometry were obtained. The detection of all analytes was carried out as [M + Na]^+^ adducts. For the remaining monosaccharides, either double peaks were present, indicating anomeric rotation and an equilibrium between two anomeric forms of the analyte in solution, or they were not detectable as sodium adducts in ESI positive mode (uronic acids), so that no reliable identification could be achieved (Fig. 1).

The limits of detection and quantification were similar in all three scanning modes (Full MS/SIM/MRM) as shown in Figure S1a, but a slight improvement was observed in SIM and MRM compared to Full MS. The LOD values obtained with Full MS mode were 2 to 5 times higher than in SIM or MRM mode except for the monosaccharide L-Rha which present the same LOD for the three scan modes (0.5 µmol/L). However, the suitability as a routine method could be considered good in all modes with relative method standard deviations less than 6% (Figure S1b).

#### HILIC-MS analysis

**Fig. 2:**
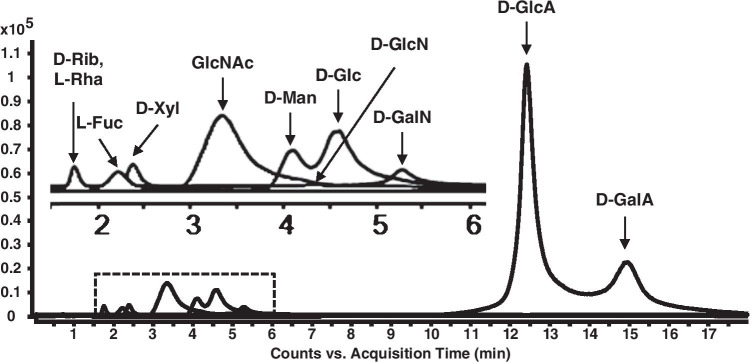
Overlaid EICs of the separable monosaccharidic species by HILIC-MS (shown here: MRM)

Application of the HILIC-MS method provided the separation and identification of eleven of the seventeen monosaccharides by either chromatography or mass spectrometry (Fig. 2). The method used here is based on the hydrophilic interaction of polar analytes with a zwitterionic stationary phase specifically developed for biological samples and modified for a wider pH range. This allows the use of a strongly basic milieu, which deprotonates the analytes and thus collapses anomers into a single product [37]. Hence, only single signals were detected for the identified substances; the remaining carbohydrates were either not separable or not accessible with MS under the given conditions.

Notable differences were found with regard to sensitivity between the various modes; SIM and MRM are in similar orders of magnitude, but are considerably more sensitive than Full MS (Figure S1c), especially for D-Glc, D-Man, GlcNAc, D-GlcA, L-Rha, L-Fuc and D-Xyl. The higher LODs found with this method for the three scan modes were observed for D-GlcN, D-GalN and D-GalA while the highest LOD was achieved for L-Rha in Full MS mode, reaching a value of 30 µmol/L (30 times higher than the LOD achieved by SIM and MRM modes). The relative method standard deviation was in some cases around 70% in Full MS, which is unacceptable for a routine method. Again, a clear improvement was observed when using SIM and MRM and was around or below 10% in these cases, which provides validity for the use as a routine method in these modes (Figure S1d).

#### RP-LC–MS analysis

**Fig. 3:**
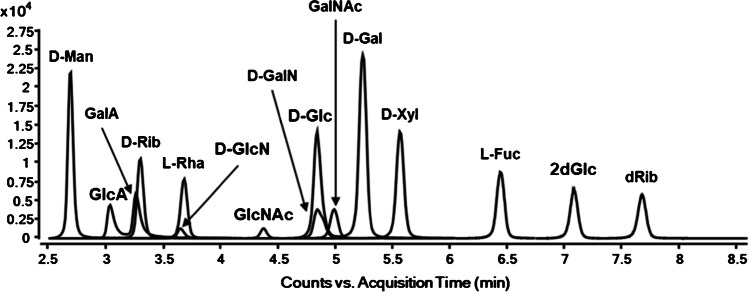
Overlaid EICs of the separable monosaccharidic species by RP-LC–MS (shown here: MRM)

Figure 3 shows the reversed-phase LC–MS analysis after derivatization, which allowed the separation of fifteen of the seventeen monosaccharides either by chromatography or mass spectrometry. The PMP groups considerably increase the retention of the polar analytes on the stationary C18 phase in comparison to the native sugars; thus, a separation was possible, with exception of the co-eluting enantiomers d- and l-glucose. In addition, arabinose could not be sufficiently separated from the isobaric xylose. Therefore, L-Glc and L-Ara were not unambiguously identifiable.

Regarding sensitivity, a strong improvement from Full MS to SIM and MRM could be observed (Figure S1e). While in Full MS, the detection limit for some analytes was still above 1 µM (D-Gal, D-GlcN, D-GalN, GlcA and GalA); for all substances in SIM and MRM, the detection limit was reached in the sub-µM range. For the relative method standard deviation, values of around or below 10% were achieved in Full MS mode. Besides the sensitivity, with SIM or MRM, a further improvement could be observed, so that RSDs below 4% were achieved, which results in a very good suitability as a routine method (Figure S1f).

#### GC–MS analysis

**Fig. 4:**
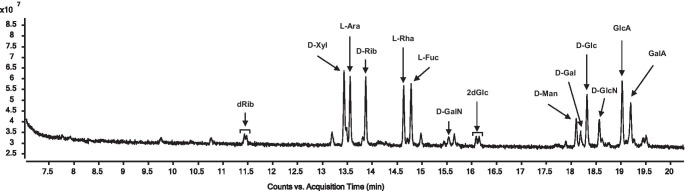
MRM chromatogram of the separable monosaccharidic species by GC–MS

Using GC–MS after derivatisation, separation of fourteen of the seventeen monosaccharides by either chromatography or mass spectrometry was achieved. As expected for GC analyses, very narrow and symmetrical signals were obtained for the individual substances (partly with double peaks, which could nevertheless be precisely assigned, compare dRib and 2dGlc in Fig. 4). As already mentioned, a total of fourteen substances could be separated; again, of course, no enantiomer separation was possible and some substances were insufficiently derivatised or were not suitable for mass spectrometry like the acetylated hexosamines.

Compared to the liquid chromatographic methods, the detection limit in the MRM mode, which was only used in the GC–MS analysis, appeared to be quite high with above 1 µM LOD in all cases. But it must be noted here that a smaller injection volume and additionally a split were utilized and by using a large volume injector an improvement can be expected (Figure S1g). In addition, excellent reproducibility was also achieved with RSDs of less than 4% (Figure S1h).

### Comparison of chromatographic methods

**Fig. 5:**
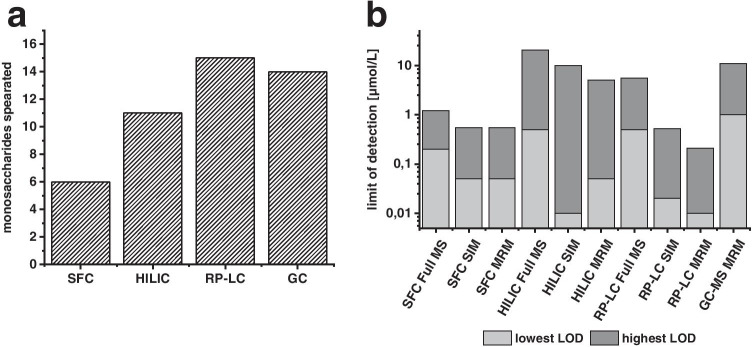
**a** Number of monosaccharides separated by chromatography or mass using the corresponding method and **b** lowest (light) and highest (dark) limits of detection for the different methods

Figure 5a shows that the separation performance of RP-LC- and GC–MS was superior to SFC- and HILIC-MS, because 15 and 14 of the 17 monosaccharides, respectively, could be separated either by chromatography or mass spectrometry and thus could be identified unambiguously. The difference in the separation power between RP-LC–MS and GC–MS was that the two N-acetylated sugars analysed (GlcNAc and GalNAc) were only separated and detected by RP-LC–MS, while L-Ara was only separated and detected by the GC–MS method. SFC and HILIC were by far inferior in the separation of monosaccharides, especially the coupling of SFC with mass spectrometry showed only insufficient separation performance. Since the gradient was already very flat, the possibility of optimizing the separation of these compounds was not given and a fundamentally different approach should rather be chosen with regard to the stationary phase or the eluent system for an improvement of the separation, which might cause more elaborative sample preparation or use of more advanced phase systems. A major problem in the SFC analysis was also the presence of two or more anomeric peaks for a single standard used. This problem can be circumvented with HILIC by using a high pH value, resulting in only one signal per substance. This eliminates the need for a preceding chemical reaction prior to chromatography, such as derivatisation. Overall, SFC and HILIC required a notably shorter sample preparation time compared to RP-LC and GC, but this is obviously at the expense of separation performance. Therefore, these methods are only suitable for a targeted analysis of the separable monosaccharides mentioned above. Further information about the retention times and detected ions in MS can be found in Table S1.

Comparatively, for all four chromatographic methods, regardless of the scanning mode of mass spectrometry, the achieved detection limits were in the low micromolar range or below, depending on the substance. For the liquid chromatographic methods, a partly considerable improvement in sensitivity can be observed when SIM or MRM was applied; the corresponding repeatability of the methods was also improved, using the relative method standard deviation as a criterion. For HILIC-MS in particular, there are a lot of interference in full MS, which leads to RSDs between 15 and 70%. Here, mass spectrometry should definitely be operated in SIM or MRM.

As already described above, a lower injection volume was selected for GC–MS than for the liquid chromatographic methods and, in addition, a split injection was applied. Besides, further sample preparation steps or additional instrumentation like programmable temperature vaporizing (PTV) or another injection method such as solid-phase microextraction (SPME) or in-tube extraction methods (iTEX) could improve the sensitivity of monosaccharides in GC–MS, which on the other hand would increase the complexity of the method.

With the available equipment, the RP-LC–MS method provides the best results for the analysis of carbohydrates in biological and food samples, both in terms of separation performance and sensitivity, with detection limits of between 10 and 200 nmol/L achieved in MRM, which corresponds to 1–39 µg/L in mass concentration. More detailed information about the analytical performance of the methods can be found in Tables S2 to S5. The separation power and sensitivity of this method was utilised to screen sugar profiles of biological and food samples.

### Application

By applying the RP-LC–MS method for the analysis of herbal liquors, plant pectins and a glycoprotein, the sugar composition and profiles of these food and biological samples were obtained. To ensure that application of the method is as accurate as possible, additional instrumental validation parameters were determined for RP-LC–MS in MRM, which can be found in Table S6. The intraday precision was found to be excellent in both the low and medium concentration ranges with RSDs in terms of peak area of triplicates below 10% for the low concentration range and below 3% for the medium concentration range. Furthermore, for these two ranges, the accuracy was also excellent with deviations from the theoretical concentration of around or below 10% for the low range and around or below 5% for the medium range. In addition, the method displays outstanding retention time stability across all calibration levels with mean deviation of 0.01 to 0.02 min. However, interday precision in relation to the peak area is low with RSDs up to 67%. Interday precision may be improved by utilising a labelled internal standard, but as external calibration was applied here, it is recommended and necessary to perform a daily calibration upon quantification of real samples.

#### Herbal liquors

Herbal liquors are containing free sugars, as well as glycosidically bound sugars. Therefore, the liquors were fractionated using SPE, to obtain a free sugars fraction (fraction 1) and a fraction containing the bound sugars (fraction 2).

It is noticeable that especially the herbal liquors’ first fraction (loading step of the SPE) contains very high concentrations of saccharidic building blocks, which are originally either monomeric or oligo- and polymeric (due to hydrolysis to monosaccharides, a distinction is not possible). The main constituents of the carbohydrates in this fraction were D-Glc and D-GalN, which is not unexpected since many herbal liquors use caramel colouring, which is produced from glucose syrup by heating with sulphuric acid and ammonia [38–40]. In all samples of this first fraction, D-GlcN could also be identified and quantified, but with a notably less abundance. Since it is well-known in literature that acetylated hexosamines are deacetylated in an acidic milieu and are subsequently present as amino sugars, such as GlcNAc into D-GlcN [41], a semi-quantitative study was carried out to evaluate the rate of deacetylation under the given hydrolysis conditions with the above-listed standards. Results showed a complete deacetylation (100%) of GalNAc to D-GalN and 98% of GlcNAc to D-GlcN during acid hydrolysis with TFA at 95 °C for 3 h (data not shown). Thus, it can be assumed that a certain part of the detected amino sugars originates from the originally acetylated form. In this free sugars fraction, however, the liquors UG and BN showed certain differences from the otherwise quite similar samples. In addition to the three main components (D-Glc, D-GalN and D-GlcN), D-Xyl, D-Gal and D-GalA were also found in UG and D-GalA in BN (Figure S2). Overall, the UG sample stood out because the dry residue was unusually low compared to the other liquors. It is suspected that in this particular herbal liquor, the colouring comes only from the extracted herbs and that subsequent colouring with caramel was largely dispensed with which reduces the amount of added free sugars as well as promotes a different composition of the free sugars since only the native sugars from the plants are present in the liquor. Therefore, the residue could be taken up in considerably less solvent, resulting in a higher concentration of the substances to be analysed. In some cases, further unknown monosaccharides were found whose retention times did not correspond to those established in the method. In general, these isomers were very low abundant signals, so that it can be assumed that they are not relevant substances. For the free sugars fraction, only in the samples of JR, KG, BN and KH an additional hexose (*m/z* 511.1, RT = 3.9 min) and in KH, SN and AA an additional hexosamine (*m/z* 510.5, RT = 3.45 min) were present in a notable level.

Fraction 2 (eluted fraction from the SPE), corresponding to hydrolysed sugars originally glycosidically bound to non-polar organic compounds, showed a remarkably lower total concentration of sugars, but overall more variation in sugar profiles (Figure S3). D-Glc and D-GalN were again present as the main constituents, but depending on the sample, up to five other monosaccharide species could be identified and quantified. Again, sample UG stands out, in which D-Xyl, L-Rha, D-GlcN, D-GlcA and D-GalA were found. In addition, as in the case of the free sugars fraction, also sample BN showed a richer sugar profile in fraction 2 than the other liquors. Overall, the more complex sugar profile in fraction 2 can also be explained by the fact that the dilution factor in the sample preparation was relatively low due to the reduced dry matter; and thus, very low abundant monosaccharides can also be found and quantitatively determined. Besides, as in the case of the free sugars fraction, unknown monosaccharide species that do not correspond to the retention time of the standards used in the method development were also found in this fraction. A particularly abundant pentose (*m/z* 481.4, RT = 4.4 min) was additionally found in sample UG. Substantially less abundant, but still notable, was an additional hexose (*m/z* 511.1, RT = 3.3) found in samples BN, KH, SN and AA. The concentrations of the detected carbohydrates in both fractions, as well as the amount of carbohydrates found in the total dry matter from 1 mL of the eight liquors, are listed in Table S7 and Table S8, respectively. The analysis of the sugar composition of herbal liqueurs or other samples rich in phenolic compounds can be of great importance for the identification of the phenolic compound profile, since many of the phenolic compounds contained in this kind of samples present sugar-related isomers, mainly glucoside and galactoside compounds. With the analysis proposed in this work, it would be very easy to determine which kind of isomer is detected in the herbal liquor samples since in this case, in none of fraction 2 obtained from the eight herbal liquors, D-Gal was detected, which means that there were no galactoside phenolic compounds present in the samples. Besides, in the samples JR, KG and UG, the only hexose isomer that was detected was D-Glc, which means that all the hexoside phenolic compounds can be identified as glucose derivatives in these samples. On the other hand, D-GlcN was detected in very low concentrations or even not detected in these fractions while a considerable concentration of D-GalN (100–278 µg/mL) was quantified. In this case, it would be possible to establish the galactosamine phenolic compounds as the isomers present in the phenolic compound profile of the herbal liquors.

When considering the results, matrix effects must of course not be disregarded. During hydrolysis, matrix components are often formed that can have different influences on the derivatisation efficiency on the one hand and on the other hand on the recovery of the individual derivatised species in the analysis via LC–MS. The matrix effects of TFA hydrolysis have already been studied by Rühmann et al. [19] concluding that for monosaccharides, the hydrolysis matrix of TFA only has a reducing influence on N-acetylated species with regard to recovery. However, since acetylated species are deacetylated during hydrolysis anyway and are subsequently present as the corresponding amino sugar, this effect is negligible here. For all other monosaccharide species, however, the hydrolysis matrix has either no significant influence or an enhancing effect. Of course, matrix effects from sample components must not be dismissed; for the sample preparation of the herbal liquors, solid-phase extraction was used, which, in addition to the enrichment of individual substances, also has a purifying effect. Thus, we assume that the two fractions obtained from the SPE are largely clean; and thus, no negative matrix effects from other sample components are to be expected.

#### Fruit pectins

Pectins are used as a vegan alternative to animal gelatines. Pectins are polysaccharides consisting mainly of a galacturonic acid backbone, which may be interrupted by other monosaccharidic units or carry further species as side chains [42, 43]. Pectins are part of the primary cell wall or the middle lamellae of plants and are obtained from different plant sources [44].

**Fig. 6:**
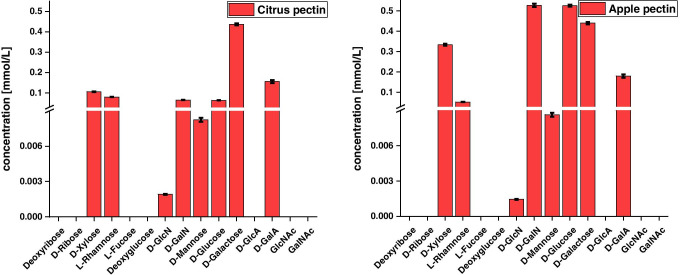
Sugar profiles of two pectins from different botanical sources (citrus left, apple right) determined by LC–MS (*n* = 3)

The two fruit pectins also show some differences in their monosaccharide composition. First of all, the total concentrations of the saccharides found and quantified differed, which may have several reasons. Firstly, a pectin consists mainly of a galacturonic acid backbone; especially in the case of acidic polysaccharides, the hydrolysis method may have to be adjusted and optimised in order to achieve the highest possible yield during hydrolysis [45]. However, since the optimisation of the hydrolysis was not the focus of this study, a hydrolysis method was chosen that is suitable on average for all polysaccharides and glycosidic bonds and is not too time-consuming. To a certain extent, the polysaccharide could be hydrolysed since D-GalA was detected. However, the carboxyl and hydroxyl groups of this polysaccharide can be esterified. If the esterification is not cleaved by the hydrolysis and the esterified groups are reduced to their original monosaccharide form, they are not detectable by our targeted LC–MS method. This explains why, overall, the amount of pectin originally used could not be recovered, assuming that the samples purchased are largely pure and contain only the pectin. Overall, about twice the amount of carbohydrates was recovered for the apple pectin than that for the citrus pectin, so that the binding patterns in the two samples obviously differed remarkably. Furthermore, it is particularly noticeable in the sugar profiles, shown in Fig. 6, that the apple pectin was clearly dominated by D-Glc and D-GalN in addition to the obvious D-GalA compared to the citrus pectin, even when the higher recovery is taken into account. Furthermore, the proportion of D-Xyl was increased here. In the other sugars found, the two pectins differed less and were largely similar.

#### Human glycoprotein

The glycoprotein, α1-acid glycoprotein (AGP) also called orosomucoid (ORM), is primarily produced in the liver, but also partly in other extrahepatic tissues, and acts as an acute phase protein that provides important functions in tissue damage and diseases, among other things by inducing immune reactions and transporting drugs [46]. AGP carries a quite high amount of carbohydrate moieties which additionally can occur in various glycoforms, including hexoses, hexosamines and deoxysugars [47].

**Fig. 7:**
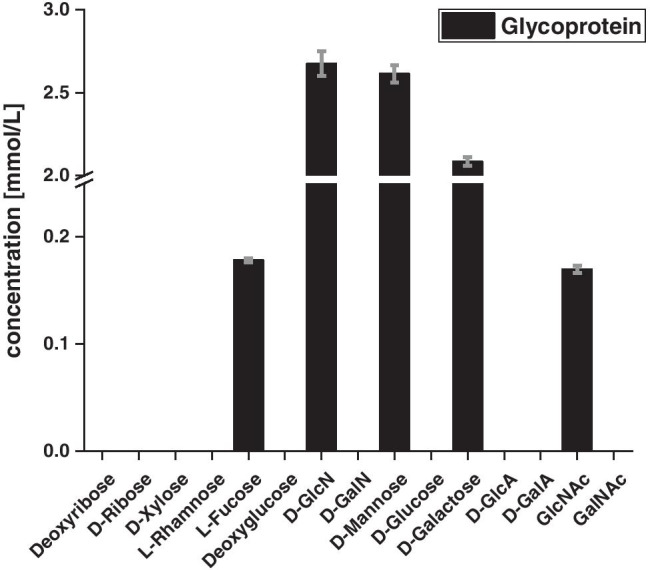
Sugar profile of the α1-acid glycoprotein (AGP) from human plasma determined by LC–MS (*n* = 3)

The glycoprotein was found to have a total carbohydrate concentration of 1.39 mg/mL, which corresponds to a sugar content of 13.9% with an initial sample concentration of 10 mg/mL. The carbohydrate moieties identified here correspond to L-Fuc, D-GlcN, D-Man, D-Gal and GlcNAc, with D-GlcN, D-Man and D-Gal dominating the sugar profile with tenfold higher concentrations than the less abundant species (Fig. 7). Overall, the glycoprotein displays a quite high amount of carbohydrates, which indicates that the polypeptide chain is highly glycosylated, which has also been reported before [48]. Therefore, acid hydrolysis seems to result in an explicitly higher yield when compared to pectins in the case of this glycoprotein.

## Conclusion

Here we compared the carbohydrate analysis using SFC, HILIC, RP-LC and GC combined with triple quadrupole mass spectrometric detection. Overall, RP-LC–MS in MRM mode after derivatisation with PMP proved to be the most suitable method in terms of separation performance, sensitivity and repeatability for the analysis of monosaccharides, with LODs between 10 and 200 nmol/L or 1 to 39 µg/L in terms of mass concentration. The applicability of this method to different biological samples was examined, in this case fractionated herbal liquors, pectins and a human glycoprotein. For all samples, prior acid hydrolysis was performed to cleave any glycosidic bonds present in polysaccharides, glycoproteins or flavonoids. For herbal liquors, sugar profiles could be obtained, which showed little variation for the fraction of “free” sugars in the liquors, the profile being dominated here by D-Glc, D-GlcN and D-GalN, with one exceptional case showing a richer profile in which up to three further monosaccharides could be identified and quantified. The second fraction from herbal liquors showed considerably more diverse profiles; here, the sugars are glycosidically bound to more non-polar molecules, such as polyphenolic compounds. In these fractions, D-Xyl, L-Rha, D-GalN, D-Man, D-Glc and D-GlcA as well as D-GalA were detected, which helps in the identification of the different phenolic compound isomers. Moreover, the comparison of two pectins from different sources (apple and citrus fruit) showed only minor differences in the profiles, but an overall higher concentration of monosaccharides was found in the apple pectin sample, which can be attributed to different esterification of the structural units between the two pectin sources or a higher hydrolysis yield. Overall, it can be stated that the hydrolysis requires optimisation depending on the sample, but since the focus here was on the method comparison on the analytical aspect and the general applicability to different biological samples and not on the optimisation of the sample preparation, this should not diminish the proof of concept. The applicability to a human glycoprotein was also successful and monosaccharides already known from the literature could be identified as glycosylation of the peptide backbone. In total, the separation performance and sensitivity were tested for 17 frequently occurring monosaccharide species, which of course does not cover the full range of carbohydrates. The methodology could still be extended to other monosaccharides, which may require further adaptation of the chromatographic method or the introduction of an additional separation dimension in the case of substances that cannot be separated either by retention time or mass difference. Here, either comprehensive two-dimensional liquid chromatography or ion mobility-mass spectrometry would be suitable, which showd a remarkable increase in separation power compared to one-dimensional methods, but on the other hand also leads to an increasing methodical challenge and thus to more complex data evaluation. Nevertheless, in this comparative study, a direct comparison of typically applied methods for the analysis of saccharides could be shown with explicit findings. The found method is also suitable for further areas of application that go beyond the real samples shown here.

## Supplementary Information

Below is the link to the electronic supplementary material.

Supplementary file1 (DOCX 1413 KB)

## Data Availability

Not applicable.
