# Supplementary material for: Comparative study for analysis of carbohydrates in biological samples
Source: Anal Bioanal Chem. 2021 Dec 20;414(6):2117–30. doi: 10.1007/s00216-021-03845-z (PMC8821481; doi:10.1007/s00216-021-03845-z)
Supplement: Supplementary file 1 — Supplementary file1 (DOCX 1413 KB) [file 216_2021_3845_MOESM1_ESM.docx]

**Supplementary Material S1 for:**

**Comparative study for analysis of carbohydrates in biological samples**

Martin Meyer^a,b^, Lidia Montero^a,b^_,_ Sven W. Meckelmann^a,b^, Oliver J. Schmitz^a,b*^

^a^Applied Analytical Chemistry, University of Duisburg-Essen, Universitaetsstrasse 5, Essen 45141, Germany

^b^Teaching and Research Center for Separation, University of Duisburg-Essen, Universitaetsstrasse 5, Essen 45141, Germany

*corresponding author

E-mail address: oliver.schmitz@uni-due.de

ORCID:

M. Meyer: 0000-0002-7360-1849

L. Montero: 0000-0001-5579-2883

S.W. Meckelmann: 0000-0002-0407-7879

O.J. Schmitz: 0000-0003-2184-1207

**a**

**b**

**d**

**f**

**h**

**c**

**e**

**g**

**Figure S1:** LODs and RSDs for separable carbohydrates with different chromatographic techniques coupled to mass spectrometry: SFC-MS (**a**, **b**), HILIC-MS (**c**, **d**), RP-LC-MS (**e**, **f**) and GC-MS (**g**, **h**); changing from Full MS to more selective MS methods, as SIM and MRM generally improved sensitivity in terms of limit of detection and the repeatability.

**Figure S2**: Sugar profiles of free sugars fraction of eight different herbal liquors determined by RP-LC-MS (n=3).

**Figure S3:** Sugar profiles of glycosidic bond sugars fraction of eight different herbal liquors determined by RP-LC-MS (n=3).

**Table S1**: Retention times, detected ions, and transitions of the screened carbohydrate standards (abbreviations *abbr.* and symbol nomenclature for glycans *SNFG* according to National Center for Biotechnology Information *NCBI*). The precursor ions also correspond to the detected (molecular ions) in full MS and SIM (n.s.=not separated, n.d.=not detected).

|  |  |  | SFC-MS | | | HILIC-MS | | | RP-LC-MS | | | GC-MS | | |
| --- | --- | --- | --- | --- | --- | --- | --- | --- | --- | --- | --- | --- | --- | --- |
| Carbohydrate | Abbr. | SNFG | RT [min] | Precursor ion [*m/z*] | Transition [*m/z*] | RT [min] | Precursor ion [*m/z*] | Transition [*m/z*] | RT [min] | Precursor ion [*m/z*] | Transition [*m/z*] | RT [min] | Precursor ion [*m/z*] | Transition [*m/z*] |
| 2-deoxy-D-glucose | 2dGlc |  | 5.2 | 187.1 | 187.1 | n.s. |  |  | 7.12 | 495.5 | 175.2 | 15.65 | 146.8 | 73.0 |
| 2-deoxy-D-ribose | dRib |  | n.s. |  |  | n.d. |  |  | 7.71 | 465.5 | 175.1 | 11.43 | 146.6 | 73.0 |
| D-galactosamine | GalN | 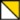 | n.s. |  |  | 5.24 | 178.1 | 89.0 | 4.91 | 510.5 | 175.1 | 15.54 | 146.7 | 73.0 |
| D-galactose | D-Gal | 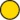 | n.s. |  |  | n.s. | 179.1 | 89.0 | 5.29 | 511.1 | 175.1 | 18.19 | 146.7 | 73.0 |
| D-galacturonic acid | GalA | 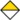 | n.d. |  |  | 14.87 | 193.0 | 89.0 | 3.36 | 525.4 | 175.1 | 19.20 | 146.7 | 73.0 |
| D-glucosamine | GlcN | 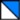 | n.s. |  |  | 4.25 | 178.1 | 89.0 | 3.66 | 510.5 | 175.1 | 18.57 | 146.7 | 73.0 |
| D-glucose | D-Glc | 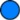 | 7.5 | 203.1 | 203.1 | 4.63 | 179.1 | 89.0 | 4.89 | 511.1 | 175.2 | 18.32 | 146.7 | 73.0 |
| D-glucuronic acid | GlcA | 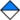 | n.d. |  |  | 12.36 | 193.0 | 89.0 | 3.12 | 525.4 | 175.1 | 19.02 | 146.7 | 73.0 |
| D-mannose | D-Man | 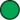 | 6.7 | 203.1 | 203.1 | 4.12 | 179.1 | 89.0 | 2.70 | 511.1 | 175.1 | 18.10 | 146.7 | 73.0 |
| D-ribose | D-Rib | 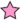 | 4 | 173.0 | 173.0 | 1.73 | 149.0 | 89.0 | 3.31 | 481.5 | 175.1 | 13.87 | 102.8 | 73.0 |
| D-xylose | D-Xyl | 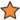 | n.s. |  |  | 2.37 | 149.0 | 89.0 | 5.59 | 481.4 | 175.2 | 13.43 | 102.8 | 73.0 |
| L-arabinose | L-Ara | 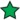 | n.s. |  |  | n.s. |  |  | n.s. |  | 175.1 | 13.56 | 102.8 | 73.0 |
| L-fucose | L-Fuc | 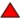 | n.s. |  |  | 2.19 | 163.1 | 89.0 | 6.48 | 495.5 | 175.1 | 14.78 | 116.8 | 73.0 |
| L-glucose | L-Glc |  | n.s. |  |  | n.s. |  |  | n.s. |  | 175.1 | n.s. |  |  |
| L-rhamnose | L-Rha | 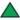 | 4.7 | 187.1 | 187.1 | 1.73 | 163.1 | 89.0 | 3.71 | 495.5 | 175.1 | 14.63 | 116.8 | 73.0 |
| N-acetyl-D-galactosamine | GalNAc | 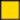 | n.s. |  |  | n.s. |  |  | 5.07 | 552.5 | 175.2 | n.d. |  |  |
| N-acetyl-D-glucosamine | GlcNAc | 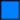 | 8.9 | 244.1 | 244.1 | 3.13 | 220.1 | 89.0 | 4.4 | 552.5 | 175.0 | n.d. |  |  |

| Carbohydrate | Abbr. | SNFG | SFC Full MS | | | | SFC SIM | | | | SFC MRM | | | |
| --- | --- | --- | --- | --- | --- | --- | --- | --- | --- | --- | --- | --- | --- | --- |
|  |  |  | LOD  [µmol/L] | LOQ [µmol/L] | r^2^ | mean RSD [%] | LOD [µmol/L] | LOQ [µmol/L] | r^2^ | mean RSD [%] | LOD [µmol/L] | LOQ [µmol/L] | r^2^ | mean RSD [%] |
| 2-deoxy-D-glucose | 2dGlc |  | 0.2 | 1 | 0.9876 | 4.5 | 0.1 | 0.5 | 0.9649 | 3.8 | 0.1 | 0.5 | 0.9742 | 5.7 |
| 2-deoxy-D-ribose | dRib |  | n.s. |  |  |  | n.s. |  |  |  | n.s. |  |  |  |
| D-galactosamine | GalN | 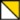 | n.s. |  |  |  | n.s. |  |  |  | n.s. |  |  |  |
| D-galactose | D-Gal | 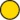 | n.s. |  |  |  | n.s. |  |  |  | n.s. |  |  |  |
| D-galacturonic acid | GalA | 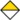 | n.d. |  |  |  | n.d. |  |  |  | n.d. |  |  |  |
| D-glucosamine | GlcN | 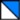 | n.s. |  |  |  | n.s. |  |  |  | n.s. |  |  |  |
| D-glucose | D-Glc | 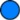 | 0.5 | 2 | 0.9961 | 5.4 | 0.1 | 1 | 0.9406 | 2.9 | 0.1 | 1 | 0.9271 | 3.4 |
| D-glucuronic acid | GlcA | 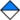 | n.d. |  |  |  | n.d. |  |  |  | n.d. |  |  |  |
| D-mannose | D-Man | 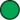 | 0.5 | 2 | 0.9901 | 4.7 | 0.1 | 0.5 | 0.9438 | 4.0 | 0.2 | 1 | 0.9642 | 3.8 |
| D-ribose | D-Rib | 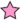 | 0.5 | 1 | 0.9921 | 3.0 | 0.2 | 1 | 0.9646 | 3.2 | 0.2 | 1 | 0.9803 | 3.5 |
| D-xylose | D-Xyl | 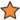 | n.s. |  |  |  | n.s. |  |  |  | n.s. |  |  |  |
| L-arabinose | L-Ara | 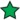 | n.s. |  |  |  | n.s. |  |  |  | n.s. |  |  |  |
| L-fucose | L-Fuc | 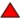 | n.s. |  |  |  | n.s. |  |  |  | n.s. |  |  |  |
| L-glucose | L-Glc |  | n.s. |  |  |  | n.s. |  |  |  | n.s. |  |  |  |
| L-rhamnose | L-Rha | 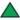 | 0.5 | 1 | 0.9943 | 2.7 | 0.5 | 1 | 0.9636 | 4.9 | 0.5 | 2 | 0.9896 | 3.5 |
| N-acetyl-D-galactosamine | GalNAc | 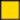 | n.s. |  |  |  | n.s. |  |  |  | n.s. |  |  |  |
| N-acetyl-D-glucosamine | GlcNAc | 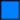 | 0.2 | 0.5 | 0.9961 | 3.4 | 0.1 | 0.5 | 0.9722 | 1.7 | 0.05 | 0.2 | 0.9788 | 2.3 |

**Table S2:** Analytical performance of SFC-MS in different scan modes (n.s.=not separated, n.d.=not detected). Non-separable and not detectable carbohydrate single standards (abbreviations *abbr.* and symbol nomenclature for glycans *SNFG* according to National Center for Biotechnology Information *NCBI*) were not included in the evaluation of the method performance afterwards.

| Carbohydrate | Abbr. | SNFG | HILIC Full MS | | | | HILIC SIM | | | | HILIC MRM | | | |
| --- | --- | --- | --- | --- | --- | --- | --- | --- | --- | --- | --- | --- | --- | --- |
|  |  |  | LOD  [µmol/L] | LOQ [µmol/L] | r^2^ | mean RSD [%] | LOD [µmol/L] | LOQ [µmol/L] | r^2^ | mean RSD [%] | LOD [µmol/L] | LOQ [µmol/L] | r^2^ | mean RSD [%] |
| 2-deoxy-D-glucose | 2dGlc |  | n.s. |  |  |  | n.s. |  |  |  | n.s. |  |  |  |
| 2-deoxy-D-ribose | dRib |  | n.d. |  |  |  | n.d. |  |  |  | n.d. |  |  |  |
| D-galactosamine | GalN | 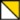 | 20 | 40 | 0.4812 | 56.5 | 2 | 5 | 0.9792 | 1.3 | 5 | 10 | 0.9476 | 8.0 |
| D-galactose | D-Gal | 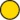 | n.s. |  |  |  | n.s. |  |  |  | n.s. |  |  |  |
| D-galacturonic acid | GalA | 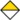 | 10 | 20 | 0.5072 | 64.3 | 5 | 10 | 0.9958 | 1.9 | 2 | 10 | 0.9891 | 4.7 |
| D-glucosamine | GlcN | 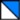 | 20 | 40 | 0.3991 | 70.3 | 10 | 40 | 0.8342 | 2.6 | 5 | 20 | 0.7415 | 8.6 |
| D-glucose | D-Glc | 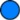 | 2 | 5 | 0.7772 | 16.3 | 0.05 | 0.5 | 0.9441 | 4.4 | 0.1 | 0.2 | 0.9838 | 5.1 |
| D-glucuronic acid | GlcA | 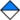 | 10 | 10 | 0.5705 | 71.2 | 0.5 | 2 | 0.9898 | 2.9 | 0.5 | 1 | 0.9845 | 4.0 |
| D-mannose | D-Man | 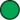 | 5 | 20 | 0.7290 | 35.4 | 0.1 | 0.5 | 0.8493 | 7.1 | 0.1 | 0.2 | 0.4281 | 11.1 |
| D-ribose | D-Rib | 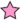 | 2 | 5 | 0.7397 | 22.4 | 0.5 | 2 | 0.9222 | 3.1 | 0.5 | 2 | 0.8846 | 7.3 |
| D-xylose | D-Xyl | 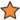 | 5 | 10 | 0.7371 | 28.2 | 0.01 | 0.5 | 0.9700 | 3.3 | 0.2 | 0.5 | 0.9517 | 5.0 |
| L-arabinose | L-Ara | 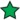 | n.s. |  |  |  | n.s. |  |  |  | n.s. |  |  |  |
| L-fucose | L-Fuc | 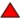 | 10 | 40 | 0.796 | 30.9 | 0.5 | 5 | 0.9780 | 3.2 | 1 | 10 | 0.8612 | 3.9 |
| L-glucose | L-Glc |  | n.s. |  |  |  | n.s. |  |  |  | n.s. |  |  |  |
| L-rhamnose | L-Rha | 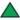 | 30 | 60 | 0.7176 | 38.1 | 1 | 5 | 0.9492 | 3.3 | 1 | 40 | 0.5405 | 9.1 |
| N-acetyl-D-galactosamine | GalNAc | 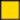 | n.s. |  |  |  | n.s. |  |  |  | n.s. |  |  |  |
| N-acetyl-D-glucosamine | GlcNAc | 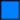 | 0.5 | 2 | 0.8351 | 14.0 | 0.05 | 0.2 | 0.9288 | 4.1 | 0.05 | 0.1 | 0.9483 | 4.7 |

**Table S3:** Analytical performance of HILIC-MS in different scan modes (n.s.=not separated, n.d.=not detected). Non-separable and not detectable carbohydrate single standards (abbreviations *abbr.* and symbol nomenclature for glycans *SNFG* according to National Center for Biotechnology Information *NCBI*) were not included in the evaluation of the method performance afterwards.

| Carbohydrate | Abbr. | SNFG | RP-LC Full MS | | | | RP-LC SIM | | | | RP-LC MRM | | | |
| --- | --- | --- | --- | --- | --- | --- | --- | --- | --- | --- | --- | --- | --- | --- |
|  |  |  | LOD  [µmol/L] | LOQ [µmol/L] | r^2^ | mean RSD [%] | LOD [µmol/L] | LOQ [µmol/L] | r^2^ | mean RSD [%] | LOD [µmol/L] | LOQ [µmol/L] | r^2^ | mean RSD [%] |
| 2-deoxy-D-glucose | 2dGlc |  | 1 | 5 | 0.9921 | 5.6 | 0.05 | 0.2 | 0.9989 | 3.0 | 0.05 | 0.2 | 0.9997 | 2.2 |
| 2-deoxy-D-ribose | dRib |  | 0.5 | 5 | 0.9942 | 3.6 | 0.05 | 0.2 | 0.9997 | 1.8 | 0.01 | 0.2 | 0.9999 | 2.3 |
| D-galactosamine | GalN | 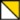 | 5 | 5 | 0.9999 | 9.9 | 0.05 | 0.2 | 0.9981 | 2.0 | 0.05 | 0.2 | 0.9989 | 2.8 |
| D-galactose | D-Gal | 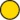 | 5 | 10 | 0.9979 | 6.4 | 0.02 | 0.1 | 0.9988 | 2.4 | 0.05 | 0.1 | 0.9992 | 2.4 |
| D-galacturonic acid | GalA | 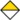 | 5 | 10 | 0.9983 | 6.3 | 0.2 | 0.5 | 0.9990 | 0.9 | 0.1 | 0.5 | 0.9986 | 2.5 |
| D-glucosamine | GlcN | 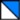 | 5 | 5 | 0.9996 | 5.9 | 0.1 | 0.2 | 0.9969 | 2.4 | 0.2 | 0.5 | 0.9972 | 3.4 |
| D-glucose | D-Glc | 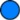 | 0.5 | 5 | 0.9997 | 9.5 | 0.02 | 0.1 | 0.9994 | 2.3 | 0.01 | 0.05 | 0.9989 | 2.6 |
| D-glucuronic acid | GlcA | 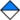 | 5 | 10 | 0.9971 | 9.6 | 0.5 | 1 | 0.9885 | 2.3 | 0.2 | 1 | 0.9905 | 2.4 |
| D-mannose | D-Man | 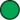 | 1 | 5 | 0.9980 | 2.8 | 0.02 | 0.1 | 0.9996 | 2.3 | 0.02 | 0.05 | 0.9996 | 2.0 |
| D-ribose | D-Rib | 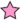 | 0.5 | 1 | 0.9991 | 10.3 | 0.05 | 0.1 | 0.9987 | 1.9 | 0.01 | 0.05 | 0.9987 | 1.9 |
| D-xylose | D-Xyl | 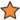 | 0.5 | 1 | 0.9989 | 3.4 | 0.05 | 0.1 | 0.9983 | 2.2 | 0.02 | 0.1 | 0.9983 | 2.9 |
| L-arabinose | L-Ara | 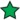 | n.s. |  |  |  | n.s. |  |  |  | n.s. |  |  |  |
| L-fucose | L-Fuc | 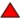 | 0.5 | 1 | 0.9925 | 5.3 | 0.05 | 0.1 | 0.9980 | 2.1 | 0.05 | 0.1 | 0.9984 | 2.4 |
| L-glucose | L-Glc |  | n.s. |  |  |  | n.s. |  |  |  | n.s. |  |  |  |
| L-rhamnose | L-Rha | 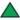 | 0.5 | 1 | 0.9924 | 3.6 | 0.05 | 0.1 | 0.9989 | 2.2 | 0.05 | 0.1 | 0.9989 | 3.3 |
| N-acetyl-D-galactosamine | GalNAc | 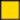 | 1 | 1 | 0.9964 | 3.8 | 0.05 | 0.1 | 0.9976 | 1.5 | 0.1 | 0.2 | 0.9991 | 3.2 |
| N-acetyl-D-glucosamine | GlcNAc | 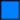 | 1 | 5 | 0.9993 | 11.3 | 0.1 | 0.5 | 0.9987 | 1.3 | 0.1 | 0.5 | 0.9991 | 2.7 |

**Table S4:** Analytical performance of RP-LC-MS in different scan modes (n.s.=not separated, n.d.=not detected). Non-separable and not detectable carbohydrate single standards (abbreviations *abbr.* and symbol nomenclature for glycans *SNFG* according to National Center for Biotechnology Information *NCBI*) were not included in the evaluation of the method performance afterwards.

**Table S5:** Analytical performance of GC-MS in MRM mode (n.s.=not separated, n.d.=not detected). Non-separable and not detectable carbohydrate single standards (abbreviations *abbr.* and symbol nomenclature for glycans *SNFG* according to National Center for Biotechnology Information *NCBI*) were not included in the evaluation of the method performance afterwards.

| Carbohydrate | Abbr. | SNFG | GC MRM | | | |
| --- | --- | --- | --- | --- | --- | --- |
|  |  |  | LOD [µmol/L] | LOQ [µmol/L] | r^2^ | mean RSD [%] |
| 2-deoxy-D-glucose | 2dGlc |  | 10 | 40 | 0.9895 | 2.8 |
| 2-deoxy-D-ribose | dRib |  | 10 | 40 | 0.9906 | 2.3 |
| D-galactosamine | GalN | 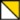 | 5 | 40 | 0.9815 | 3.4 |
| D-galactose | D-Gal | 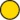 | 2 | 10 | 0.9836 | 2.6 |
| D-galacturonic acid | GalA | 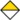 | 5 | 20 | 0.9875 | 2.7 |
| D-glucosamine | GlcN | 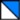 | 5 | 20 | 0.9919 | 2.4 |
| D-glucose | D-Glc | 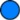 | 2 | 10 | 0.9881 | 2.4 |
| D-glucuronic acid | GlcA | 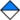 | 2 | 10 | 0.9824 | 2.5 |
| D-mannose | D-Man | 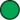 | 2 | 10 | 0.9899 | 2.0 |
| D-ribose | D-Rib | 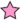 | 2 | 10 | 0.9836 | 3.3 |
| D-xylose | D-Xyl | 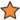 | 2 | 5 | 0.9832 | 1.9 |
| L-arabinose | L-Ara | 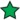 | 2 | 10 | 0.9818 | 2.9 |
| L-fucose | L-Fuc | 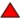 | 2 | 5 | 0.9832 | 2.2 |
| L-glucose | L-Glc |  | n.s. |  |  |  |
| L-rhamnose | L-Rha | 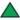 | 1 | 5 | 0.9870 | 2.4 |
| N-acetyl-D-galactosamine | GalNAc | 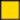 | n.d. |  |  |  |
| N-acetyl-D-glucosamine | GlcNAc | 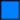 | n.d. |  |  |  |

**Table S6:** Instrumental validation parameters for RP-LC-MS in MRM mode. a) Intraday precision expressed as relative standard deviation (RSD) of obtained peak areas from triplicates analysed within the same day (n=3). Low level defined as concentration at determined limit of quantification, high level defined at concentration of 60 µmol/L. b) Interday precision expressed as relative standard deviation (RSD) of obtained peak areas from two sets of triplicates analysed with an interval of two months (n=6). Low level defined as concentration at determined limit of quantification, high level defined at concentration of 60 µmol/L. c) Accuracy calculated by comparison of ratio from theoretical concentration and concentration obtained from the calibration mathematically (n=3). d) Retention time (RT) stability calculated as RT standard deviation from triplicates of fifteen calibration levels (n=45).

| Carbohydrate | *Intraday precision*  RSD [%] | | *Interday precision*  RSD [%] | | *Accuracy*  deviation [%] | | *RT standard deviation*  [min] |
| --- | --- | --- | --- | --- | --- | --- | --- |
|  | low | high | low | high | low | high |  |
| 2-deoxy-D-glucose | 1.9 | 0.3 | 4.1 | 6.5 | 0.1 | 1.3 | 0.01 |
| 2-deoxy-D-ribose | 2.0 | 0.3 | 17.2 | 12.7 | 6.1 | 0.5 | 0.01 |
| D-galactosamine | 3.9 | 2.6 | 40.5 | 21.0 | 13.2 | 0.8 | 0.01 |
| D-galactose | 8.8 | 0.2 | 1.9 | 11.5 | 3.5 | 0.1 | 0.01 |
| D-galacturonic acid | 2.3 | 1.9 | 26.8 | 19.1 | 6.7 | 5.4 | 0.01 |
| D-glucosamine | 4.3 | 0.8 | 26.4 | 10.0 | 2.8 | 2.9 | 0.01 |
| D-glucose | 5.9 | 2.6 | 4.3 | 21.6 | 16.8 | 0.6 | 0.01 |
| D-glucuronic acid | 2.2 | 2.2 | 67.5 | 57.3 | 2.2 | 4.8 | 0.02 |
| D-mannose | 3.9 | 1.9 | 31.1 | 27.9 | 7.4 | 0.6 | 0.01 |
| D-ribose | 5.2 | 0.4 | 15.9 | 21.2 | 1.9 | 0.4 | 0.01 |
| D-xylose | 3.1 | 0.6 | 1.9 | 10.8 | 1.1 | 0.9 | 0.01 |
| L-arabinose | 2.1 | 0.7 | 7.1 | 16.8 | 2.3 | 1.9 | 0.01 |
| L-fucose | 3.3 | 0.6 | 16.3 | 19.6 | 2.4 | 3.8 | 0.01 |
| L-glucose | 1.1 | 1.2 | 15.9 | 14.0 | 1.1 | 0.3 | 0.01 |
| L-rhamnose | 4.4 | 1.2 | 40.0 | 35.3 | 0.7 | 1.1 | 0.01 |

**Table S7:** Quantitative results of the determination of sugar profiles from fractionated herbal liquors and fruit pectins with RP-LC-MS.

|  | Concentration [µg/mL] | | | | | | | | |
| --- | --- | --- | --- | --- | --- | --- | --- | --- | --- |
| Sample | Xylose | Rhamnose | Glucos-amine | Galactos-amine | Mannose | Glucose | Galactose | Glucuronic acid | Galacturonic acid |
| JR F1 | - | - | 39.4 ± 1.1 | 31489.5 ± 467.5 | - | 31589.7 ± 341.0 | - | - | - |
| JR F2 | - | 6.6 ± 0.1 | 0.5 ± 0.0 | 277.1 ± 4.1 | - | 280.0 ± 3.0 | - | - | - |
| KG F1 | - | - | 53.5 ± 1.5 | 39653.4 ± 588.7 | - | 39937.4 ± 431.1 | - | - | - |
| KG F2 | - | - | - | 102.1 ± 1.5 | - | 100.3 ± 1.1 | - | - | - |
| UG F1 | 39.9 ± 0.6 | - | 11.6 ± 0.3 | 1832.7 ± 27.2 | - | 1854.1 ± 195.1 | 1.6 ± 0.0 | - | 47.7 ± 2.1 |
| UG F2 | 12.7 ± 0.2 | 25.1 ± 0.2 | 0.4 ± 0.0 | 193.8 ± 2.9 | - | 195.1 ± 2.1 | - | 9.1 ± 0.4 | 5.5 ± 0.2 |
| BN F1 | - | - | 51.6 ± 1.4 | 46592.8 ± 691.8 | - | 47787.3 ± 515.9 | - | - | 258.2 ± 11.5 |
| BN F2 | 0.4 ± 0.0 | 62.7 ± 0.6 | - | 200.5 ± 3.0 | 5.9 ± 0.1 | 214.9 ± 2.3 | - | 2.8 ± 0.1 | 2.6 ± 0.1 |
| KH F1 | - | - | 64.4 ± 1.8 | 107245.4 ± 1592.3 | - | 108080.3 ± 1166.7 | - | - | - |
| KH F2 | - | - | - | 279.1 ± 4.1 | 14.6 ± 0.3 | 278.8 ± 3.0 | - | - | - |
| SN F1 | - | - | 44.7 ± 1.2 | 29477.5 ± 437.7 | - | 29670.8 ± 320.3 | - | - | - |
| SN F2 | - | - | - | 100.0 ± 1.5 | 8.7 ± 0.2 | 101.6 ± 1.1 | - | - | - |
| RI F1 | - | - | 54.6 ± 1.5 | 31422.1 ± 466.5 | - | 31261.7 ± 337.5 | - | - | - |
| RI F2 | - | - | 0.4 ± 0.0 | 269.4 ± 4.0 | 8.0 ± 0.2 | 265.5 ± 2.9 | - | - | - |
| AA F1 | - | - | 66.2 ± 1.8 | 40125.2 ± 595.7 | - | 40318.7 ± 435.2 | - | - | - |
| AA F2 | - | - | - | 278.1 ± 4.1 | 17.6 ± 0.3 | 273.2 ± 2.9 | - | - | 2.3 ± 0.1 |
| Citrus  pectin | 15.9 ± 0.3 | 13.1 ± 0.1 | 0.3 ± 0.0 | 11.6 ± 0.2 | 1.5 ± 0.0 | 11.4 ± 0.1 | 78.6 ± 1.0 | - | 30.3 ± 1.4 |
| Apple  pectin | 50.1 ± 0.8 | 8.6 ± 0.1 | 0.3 ± 0.0 | 94.3 ± 1.4 | 1.6 ± 0.0 | 94.6 ± 1.0 | 79.2 ± 1.0 | - | 35.0 ± 1.6 |
| Glyco-protein* | - | - | 479.3 ± 13.1 | - | 471.1 ± 9.3 | - | 375.8 ± 4.5 | - | - |

* additionally 29.2 ± 0.3 µg/mL L-Fucose and 37.6 ± 0.7 µg/mL N-Acetyl-D-glucosamine

**Table S8:** Total carbohydrate content, dry residue of 1 mL sample and saccharide percentage in total dry residue from fractionated herbal liquors, fruit pectins and α1-acid glycoprotein with RP-LC-MS stated as mg/mL.

| Sample | Sum of carbohydrate concentration [mg/mL] | Determined dry weight of 1 mL sample [mg] | Sugar content in total dry weight [%] |
| --- | --- | --- | --- |
| JR F1 | 63.12 | 160.10 | 39.4 |
| JR F2 | 0.56 | 36.20 | 1.6 |
| KG F1 | 79.64 | 193.20 | 41.2 |
| KG F2 | 0.20 | 35.00 | 0.6 |
| UG F1 | 3.79 | 42.40 | 8.9 |
| UG F2 | 0.44 | 29.80 | 1.5 |
| BN F1 | 94.69 | 247.40 | 38.3 |
| BN F2 | 0.49 | 24.30 | 2.0 |
| KH F1 | 215.39 | 373.00 | 57.7 |
| KH F2 | 0.57 | 23.40 | 2.4 |
| SN F1 | 59.19 | 175.00 | 33.8 |
| SN F2 | 0.21 | 7.80 | 2.7 |
| RI F1 | 62.74 | 250.30 | 25.1 |
| RI F2 | 0.54 | 17.20 | 3.2 |
| AA F1 | 80.51 | 247.30 | 32.6 |
| AA F2 | 0.57 | 20.80 | 2.7 |
| Citrus  pectin | 0.16 | 2.00 | 8.1 |
| Apple  pectin | 0.36 | 2.00 | 18.2 |
| Glycoprotein | 1.39 | 10.00 | 13.9 |
